# European progress in working towards a tobacco-free generation

**DOI:** 10.1007/s00431-021-04116-w

**Published:** 2021-05-25

**Authors:** Jasper V. Been, Anthony A. Laverty, Aikaterini Tsampi, Filippos T. Filippidis

**Affiliations:** 1 Division of Neonatology, Department of Pediatrics, Erasmus MC Sophia Children’s Hospital, University Medical Centre Rotterdam, Rotterdam, Netherlands; 2 Department of Obstetrics and Gynaecology, Erasmus MC Sophia Children’s Hospital, University Medical Centre Rotterdam, Rotterdam, Netherlands; 3 Department of Public Health, Erasmus MC, University Medical Centre Rotterdam, Rotterdam, Netherlands; 4 Public Health Policy Evaluation Unit, School of Public Health, Imperial College London, London, UK; 5 Department of Transboundary Legal Studies, Faculty of Law, University of Groningen, Groningen, Netherlands

**Keywords:** Child, Tobacco, Smoking, Smoke-free policy, Smoking cessation, Europe

## Abstract

Children have the right to grow up free from the hazards associated with tobacco smoking. Tobacco smoke exposure can have detrimental effects on children’s health and development, from before birth and beyond. As a result of effective tobacco control policies, European smoking rates are steadily decreasing among adults, as is the proportion of adolescents taking up smoking. Substantial variation however exists between countries, both in terms of smoking rates and regarding implementation, comprehensiveness and enforcement of policies to address smoking and second-hand smoke exposure. This is important because comprehensive tobacco control policies such as smoke-free legislation and tobacco taxation have extensively been shown to carry clear health benefits for both adults and children. Additional policies such as increasing the legal age to buy tobacco, reducing the number of outlets selling tobacco, banning tobacco display and advertising at the point-of-sale, and introducing plain packaging for tobacco products can help reduce smoking initiation by youth. At societal level, health professionals can play an important role in advocating for stronger policy measures, whereas they also clearly have a duty to address smoking and tobacco smoke exposure at the patient level. This includes providing cessation advise and referring to effective cessation services.

*Conclusion*: Framing of tobacco exposure as a child right’s issue and of comprehensive tobacco control as a tool to work towards the ultimate goal of reaching a tobacco-free generation can help accelerate European progress to curb the tobacco epidemic.

**Table:** 

**What is Known:** *• Tobacco exposure is associated with a range of adverse health effects among babies and children.* *• Comprehensive tobacco control policies helped bring down smoking rates in Europe and benefit child health.*	
**What is New:** *• Protecting the rights and health of children provides a strong starting point for tobacco control advocacy.* *• The tobacco-free generation concept helps policy-makers set clear goals for protecting future generations from tobacco-associated harms.*

**What is Known:**

*• Tobacco exposure is associated with a range of adverse health effects among babies and children.*

*• Comprehensive tobacco control policies helped bring down smoking rates in Europe and benefit child health.*

**What is New:**

*• Protecting the rights and health of children provides a strong starting point for tobacco control advocacy.*

*• The tobacco-free generation concept helps policy-makers set clear goals for protecting future generations from tobacco-associated harms.*

## Synopsis

Tobacco smoke exposure poses a range of significant risks to child health and violates child rights [1, 2]. While the health impacts of smoking for children generally receive less attention than those for adults, they constitute a substantial cause of mortality and morbidity in early life. Levels of tobacco exposure have come down in recent years in Europe, driven by robust policy action at local, national and regional levels. However, the serious related harms still evident mean that we have further to go to achieve a truly tobacco-free generation. Required actions including expanding smoke-free legislation, increases in tax and price and more consistently delivering interventions within health care settings are all warranted.

## How tobacco affects children

There are a number of pathways through which children are affected by tobacco smoking. Before birth exposure can occur through either active smoking or second-hand smoke (SHS) exposure of pregnant women, while growing up children can be exposed to SHS for example by smoking within the family, and in later childhood they may take up smoking themselves. Each of these pathways harms children in different ways. In Europe, SHS exposure is linked to 170 thousand deaths every year, including 2300 deaths among children 0–14 years [3]. Smoking during pregnancy can cause serious sequelae including birth defects, preterm birth, fetal growth restriction and perinatal mortality [4, 5]. Tobacco exposure during early life furthermore increases children’s risks of sudden infant death syndrome [6], of developing respiratory tract infections (RTIs) [7], these generally being more severe, and of asthma exacerbations [8]. Emerging evidence suggests that smoking may even have impacts across generations, with for example increased risk of asthma among grandchildren of women who smoked in pregnancy [9, 10]. Exposure of mothers to SHS in pregnancy is also associated with increased risks of many of these outcomes, albeit to a lesser degree [11].

The last main pathway of children taking up smoking themselves leaves them vulnerable to the myriad health problems caused by smoking among adults [12]. Children who try tobacco at an earlier age are more likely to go on to become smoking adults [12]. Also, children living with a smoker are much more likely to try tobacco (OR 1.7 if father smokes, 2.2 if mother smokes, and 2.7 if both parents smoke), which highlights the exemplar role of parents in not smoking themselves [13]. There is additionally some evidence that third-hand smoke (THS), that is, residual smoke pollutants lingering on environmental surfaces after the smoke has cleared, may be harmful for health [14]. Studies have demonstrated increased levels of nicotine derivatives in non-smokers living in homes originally occupied by smokers [15]. THS constituents have even been found in premature babies cared for in an intensive care environment whose parents smoke, this being associated with local environmental THS contamination [16]. A wide variety of adverse health effects from THS have been observed in animal experiments [14], and the finding that children whose parents only smoke outside are still at increased risk of developing respiratory symptoms indicates that THS can also adversely impact human health [17, 18]. The different pathways through which we know that tobacco can harm children mean that we need action on reducing tobacco smoke exposure, ideally through reducing the number of adult smokers, as well as efforts to prevent taking up smoking among children themselves.

## Tobacco use in Europe: facts and figures

**Fig. 1:**
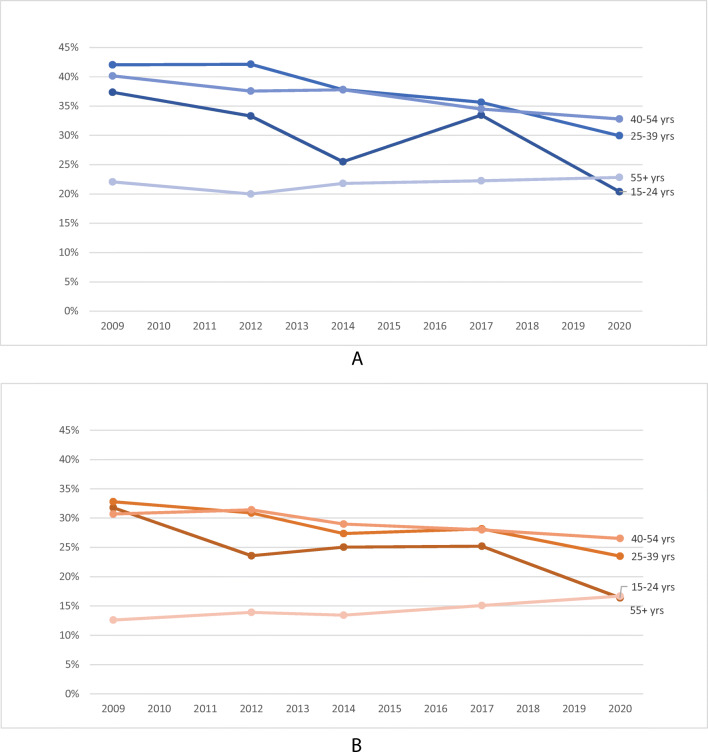
Trends in current smoking among men (**A**) and women (**B**) according to age in European Union. Data from Eurobarometer surveys waves 72.3 (2009), 77.1 (2012), 82.4 (2014), 87.1 (2017) and 93.2 (2020)

Europe is one of the world’s regions with the highest prevalence of smoking, especially among women [19]. Fortunately, the prevalence of smoking has been declining in Europe (Fig. 1) [20]. In 2020, the prevalence of smoking among people aged 15 years or older was 23% in the European Union (EU) [21], down from 29% a decade earlier [22]. However, the EU is very heterogeneous with regard to tobacco use. In countries such as Greece, Bulgaria and Croatia, more than 35% of those aged 15 years or older are current smokers, whereas the prevalence of smoking is as low as 7% in Sweden and 12% in the UK [21]. The most recent Eurobarometer survey on tobacco in 28 European countries found that 26% of men and 21% of women were current smokers in 2020 [21]. Despite the high prevalence of smoking among women in Europe, it appears that many successfully quit smoking during pregnancy without external help; however, data on this are scarce and based on selected populations that may not be nationally representative [23, 24]. Women in vulnerable groups, with low education and unplanned pregnancies, have been consistently shown to be less likely to quit smoking during pregnancy across several European countries [23, 24]. This is consistent with studies in which people facing financial problems are more likely to smoke and be exposed to SHS, which further compounds inequalities in active and passive exposure to tobacco smoke among pregnant women and young children in Europe [21]. Existing socioeconomic disparities in smoking contribute importantly to health inequalities in early life [25]. Among pregnant women, those requiring external help to quit smoking have relatively low success rates, and many restart smoking after pregnancy [26].

It has been estimated that 20% of pregnant women and 12% of children are regularly exposed to SHS at home in the EU [27], but this also varies widely between countries depending on the prevalence of smoking and the level of enforcement of smoke-free legislation. This is reflected in recent European data on exposure to SHS in restaurants. While in most central and northern European countries fewer than 5% of the respondents reported recent SHS exposure in a restaurant in 2020, there are countries like Cyprus where 40% of people who recently visited a restaurant reported being exposed to SHS [21]. Given the lingering presence of THS in environments where people smoke or where smokers are [14–16], the proportion of women and children exposed to THS is likely even higher. The picture is further complicated by the recent rise in popularity of e-cigarettes and heated tobacco products. Although current use of these products is not widespread, they are quite popular in certain countries and among younger ages, including women of childbearing age [21]. Studies in Germany and the UK have shown that some women may continue to use e-cigarettes during pregnancy or start using them to quit smoking [28, 29]. The clinical implications of this are now only starting to be explored [30].

Regarding smoking uptake, a European survey in 2017/2018 showed that, at age 11, 5% of boys and 2% of girls had ever smoked, this increasing to 29% and 27%, respectively, at age 15 [31]. Rates had improved slightly compared to 2014, but there were substantial between-country differences, with Bulgaria, Lithuania and Italy having particularly high adolescent smoking rates [31]. A recent European survey confirmed that well over half of adult smokers started smoking regularly before age 18 [32]. Also, adolescents who use e-cigarettes are more likely to become smokers in the future, although it is not entirely clear if this indicates a true gateway effect or shared risk factors [33]. Even those who do not transition to combustible tobacco use may still be exposed to health risks associated with e-cigarette use [34]. Considering the above as well as the increasing popularity of heated tobacco products among youth [21], these novel products highlight the need to expand tobacco control policies and protect children from risks associated with their use.

## Tobacco control progress in Europe

**Fig. 2:**
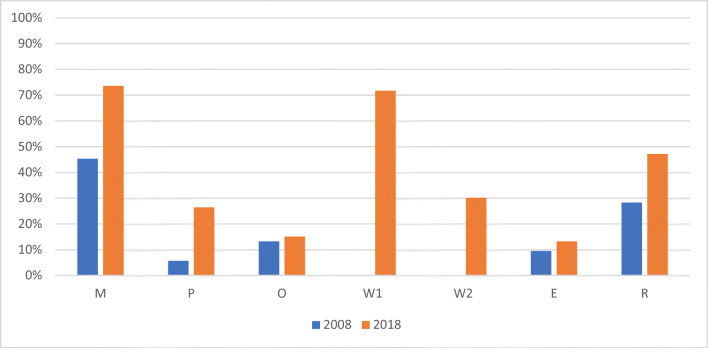
Percentage of European countries covered in 2008 and 2018 by each of the MPOWER policies at the highest level as recommended by the World Health Organization. *M* monitoring tobacco use and prevention policies, *P* protect people from tobacco smoke (i.e. smoke-free legislation), *O* offer help to quit tobacco use (i.e. access to and reimbursement of cessation services), *W* warn about the dangers of tobacco (e.g. pack warnings (W1); media campaigns (W2)), *E* enforce bans on tobacco advertising, promotion and sponsorship, *R* raise taxes on tobacco

Recent declines in smoking in Europe can be largely attributed to the aggressive tobacco control policies implemented across the continent [20]. Almost all European countries have ratified the World Health Organization (WHO) Framework Convention on Tobacco Control (FCTC), an international public health treaty launched in 2003 which outlines key evidence-based policies to curb the tobacco epidemic [35]. Close to 450 million people are also covered by the EU Tobacco Products Directive (TPD) which mandates additional tobacco control measures, such as pictorial warning labels [36]. According to the WHO, the majority of countries in the European region score highly in monitoring tobacco use, banning tobacco advertising, implementing health warnings regarding the dangers of tobacco use, taxing tobacco products and supporting smoking cessation [19], making Europe the region with the best adherence to the WHO MPOWER recommendations (Table 1; Fig. 2). Although only few countries have implemented all these policies to the highest level as recommended by the WHO, including comprehensive smoke-free legislation in public places, sustained increases in tobacco taxation, free support for smoking cessation and large pictorial warning labels [19], some have adopted additional tobacco control policies, such as smoking bans in vehicles when children are present and plain packaging. Overall, implementation is lacking in several countries, especially in areas such as smoke-free environments. Fourteen out of 36 European countries received a score below 50 (out of 100) in the most recent Tobacco Control Scale (TCS), which ranks countries based on a number of tobacco control indicators [37]. The TCS recognises the UK, France and Ireland as tobacco control leaders within Europe, with Germany, Switzerland and Luxembourg performing most poorly.

**Table 1:** MPOWER policies recommended by the World Health Organization (WHO)

Acronym	Group of tobacco control policies	Highest WHO-recommended level
M	Monitoring tobacco use and prevention policies	Recent, representative and periodic data for both adults and youth
P	Protect people from tobacco smoke	All public places completely smoke-free
O	Offer help to quit tobacco use	National quit line, and both nicotine replacement therapy and some cessation services (cost-covered)
W	Warn about the dangers of tobacco	Large warning labels on tobacco packaging with all appropriate characteristics (W1); national anti-tobacco campaign with at least seven appropriate characteristics^a^ including airing on television and/or radio (W2)
E	Enforce bans on tobacco advertising, promotion and sponsorship	Ban on all forms of direct and indirect advertising of tobacco.
R	Raise taxes on tobacco	≥ 75% of tobacco retail price is tax.

^a^The WHO outlines eight characteristics for successful mass media campaigns [14]

## Impact of tobacco control policies on children

These measures to reduce smoking among the general population can benefit children by reducing the exposure of children to SHS as well as denormalising the act of smoking. For example, smoke-free laws in public places are one of the most well-evaluated tobacco control interventions in both Europe and other high-income countries. Systematic review evidence points to reductions in preterm births, and in hospital attendance for severe RTIs and asthma hospitalisations among children from these measures [38]. These interventions can work to reduce SHS exposure in public places covered by these laws, through potential reductions in smoking prevalence in their own right, and through norm-spreading behavioural changes such as reducing smoking in the home. These impacts can be enhanced by strong mass media messaging and advertising: recent data from Scotland indicates that a novel campaign called Take It Right Outside, focused on reducing domestic SHS exposure, was successful in reducing asthma admissions among children under 5 years old [39].

Increases in prices of tobacco are also linked to improved infant survival in Europe [40], most likely through reductions in maternal smoking and SHS exposure. One study in 23 EU countries estimated that cigarette price increases between 2005 and 2014 had averted a total of over 9000 infant deaths [40]. Several countries are now moving forward by implementing novel policies including the banning of smoking inside private vehicles with children present [41]. As children do not have substantial control over their environment, and as smoking inside vehicles is linked to very high levels of toxins, these measures are justified to reduce exposure and harm. Emerging evidence now indicates that these laws can help reduce exposure to SHS among children in vehicles [42]. Nonetheless, one study pointed to low levels of enforcement of these laws jeopardising the potential health gains [43], an issue which is common across all tobacco control policies.

There are also policies with the potential to reduce the initiation of smoking among children themselves. This is crucial given that about one in two children who tries tobacco will go on to develop nicotine addiction and that over half of adult smokers commenced smoking when they were still a child [32, 44]. Effective policies include restricting the numbers of outlets selling tobacco [45], restricting the display of tobacco at point-of-sale [46, 47] and age of sale regulations [48]. Finally the introduction of plain (or standardised) packaging for tobacco has been successful in restricting the ability of the tobacco industry to use packs for advertising to lure children into using tobacco [49]. Given that early initiation of smoking is often influenced by parents and peers, an overarching impact of tobacco control efforts bringing down overall smoking rates and changing social norms is important [32].

## Addressing smoking in the health care setting

Effective tobacco control policies can make a substantial contribution to benefiting child health at the population level, as outlined [1, 50]. At the individual level, perinatal and paediatric health care providers can play a crucial role in addressing smoking as a key determinant of adverse pregnancy and child health outcomes. Enquiring about smoking status of future parents, caregivers and teenage patients should be an integral part of care. Opening subsequent discussions about the dangers of smoking and the benefits of cessation is essential. A non-judgmental, affirmative approach is important, framing the issue as a cause for concern from the child’s point of view. Professionals should realise that nicotine is highly addictive and that many additional factors may contribute to sustaining smoking, providing barriers to effective cessation even though many smokers would rather quit. Smokers should always be provided with at least a brief smoking advice and health professionals need to be conscious of national and local routes to direct them towards effective cessation support services [51, 52]. These may include national quit lines, counselling services and pharmacological support including nicotine replacement therapy. Health professionals should furthermore consider their exemplary role towards patients and the wider society in refraining from smoking themselves [53, 54].

## Tobacco as a child rights issue in Europe

Human rights and the rights of the child, in particular, are a potent tool for achieving a smoke-free generation in Europe [2, 55]. Given their vulnerability, children have, among others, the right to special protection against physical and moral dangers, such as those implied by tobacco, and the right to health, which must be guaranteed even in the private sphere. After all, the best interests of the child, a focal human rights principle, should be considered in all actions pertaining to children. The mobilisation of both the international and the European human rights law monitoring machinery can offer a significant advantage in the fight against smoking (Table 2) [56, 57]. The United Nations Convention on the Rights of the Child [58], ratified by all European States and also explicitly recalled in the Preamble of the FCTC [35], has been interpreted to imply that States have a legal obligation to protect children against the harmful effects of tobacco [2]. The same goes for the Council of Europe European Social Charter [59]. The European Committee of Social Rights, which monitors its implementation, regards smoking as one of the dangers against which children and young people should be protected and requires health education on prevention of smoking throughout the entire period of schooling as part of school curricula [60, 61]. The Committee points out that, to be effective, any prevention policy on tobacco must restrict the supply of tobacco through controls on production, distribution, advertising and pricing. In particular, the sale of tobacco to young persons must be banned [62], as must smoking in public places, including transport, and advertising on posters and in the press [63]. Clearly, these conclusions are largely consistent with WHO FCTC and TPD provisions outlined earlier [35, 36].

**Table 2:** Overview of pertinent children’s rights/interests and their indicative implications for tobacco control

Children’s rights and interests relevant to tobacco control	Indicative implications
Best interest of the child (Articles 3 CRC; 24 EU Charter)	Ensure that children’s best interests are consistently implemented in every tobacco-related action taken; ensure the child such protection and care as is necessary for his or her well-being by protecting it from tobacco
Health (Articles 24 CRC; 11 ESC; 35 EU Charter; the ECHR does not contain such a right but see Articles 2, 3, 8 ECHR and Article 2 Protocol no 1 to the ECHR)	Take appropriate measures to reduce the use of tobacco among children; prevent exposure to smoking; provide advisory and educational facilities for the promotion of health and education in schools on prevention of smoking; restrict the supply of tobacco through controls on production, distribution, advertising and pricing; ban the sale of tobacco to young persons; create smoke-free spaces; ban tobacco advertising
Life [survival and development] (Articles 6 CRC; 2 ECHR; 2 EU Charter)	As above
Information (Articles 17 CRC; 11 EU Charter)	Provide appropriate information to protect children against the harmful effects of tobacco
Government support for parental responsibility to protect best interest of the child (Article 18 CRC)	Support parents to avoid exposing children to environmental tobacco smoke
Protection against physical and moral dangers (Articles 19 CRC; 7.10 ESC)	Protection against the spread of smoking
Adequate standard of living (Article 27 CRC)/Respect for private and family life (Articles 8 ECHR; 7 EU Charter)	Ensure children are not exposed to tobacco smoke inside their homes
Protection against exploitation (Article 36 CRC)	Protect children against tobacco marketing

*CRC* United Nations Convention on the Rights of the Child [58], *EU* European Union, *ESC* Council of Europe European Social Charter [59], *ECHR* European Convention on Human Rights [65]

These observations show the potentials of framing tobacco as a child rights issue in Europe. The deployment of human rights–based arguments and human rights language, with emphasis on the rights of the child and its best interests, can be significant in awareness raising, policy-making and litigation to guarantee a tobacco-free generation. These potentials can be further untapped especially within the Council of Europe human rights system [64], through the promotion of the existing standards and the integration of tobacco control in the work of all the Council of Europe’s relevant organs. The more systematic invocation of these standards by individuals (e.g. children, parents) and entities (e.g. advocacy groups, state authorities) implicated in the fight against tobacco to protect children’s health can be an additional path towards this direction. The European Convention on Human Rights and the EU Charter of Fundamental Rights can also be further used in this context [65–68].

## European initiatives for a tobacco-free generation

The framing of tobacco use as a child rights issue and emphasising the need to protect children from the harms of tobacco are clearly gaining momentum as a starting point for advocacy for stronger tobacco control policies [69]. An increasing number of countries have set specific targets for working towards attaining a smoke-free (or tobacco-free) generation by a fixed target date, framing their tobacco control strategy around this concept. Variation exists in such goalsetting, whether aiming for overall smoking rates to go below a certain level or for smoking initiation among youth to drop to zero, or whether going for smoke-free, tobacco-free (i.e. including non-combustible tobacco) or nicotine-free (i.e. including e-cigarettes). England for example in their tobacco control plan for 2017–2022 expressed the ambition to reduce regular smoking among 15-year-olds from 8 to ≤ 3% and reduce smoking during pregnancy from 10.7 to ≤ 6% [70]. In the Netherlands, the smoke-free generation concept was a key driver of goalsetting within the National Prevention Agreement signed by the national government and over 70 societal organisations in 2019 [71]. In addition to governmental policies, the Agreement specifies targets and responsibilities for signatories including for example ensuring that the grounds of petting zoos, sports associations and health care institutions will become smoke-free. Extension of smoke-free policies to include outdoor areas and private areas such as cars, implementing of plain packaging and restricting the point-of-sale display of tobacco products, and reducing the number of outlets selling tobacco are among the key policies included in many national tobacco-free generation strategies, with the overall aim to denormalise smoking [1, 44, 70, 71].

## Conclusion

Children have the right to grow up free from the substantial hazards associated with tobacco use and they need to be acknowledged as important stakeholders in its regulation. Strong tobacco control policies are associated with clear population health benefits, including among children. Although European smoking rates are relatively high, particularly among women, they are decreasing steadily under influence of effective policy-making. Substantial between-country variation however exists and much more can and still needs to be done to work towards reaching the ultimate goal of attaining a tobacco-free generation.

## Data Availability

Not applicable.

## Abbreviations

FCTC: Framework Convention on Tobacco Control
EU: European Union
RTI: Respiratory tract infection
SHS: Second-hand smoke
TCS: Tobacco control scale
THS: Third-hand smoke
TPD: Tobacco Products Directive
WHO: World Health Organization
